# Comparison of efficacy of bupivacaine and fentanyl with bupivacaine and sufentanil for epidural labor analgesia

**DOI:** 10.4103/1658-354X.71569

**Published:** 2010

**Authors:** Sumit Kalra, Namita Saraswat, G. S. Agnihotri

**Affiliations:** *Department of Anaesthesia, Lady Hardinge Medical College, New Delhi, India*; 1 *Department of Anaesthesia, Ghandi Medical College, Bhopal, India*

**Keywords:** *Labor analgesia*, *bupivacaine fentanyl*, *sufentanil*, *epidural analgesia*

## Abstract

**Objectives:**

A study to compare the efficacy between fentanyl and sufentanil combined with low concentration (0.0625%) of bupivacaine for epidural labor analgesia in laboring women

**Materials and Methods:**

Fifty full term parturients received an initial bolus dose of a 10 ml solution containing 0.125% bupivacaine. The patients were randomly divided into two: group F received 0.0625% bupivacaine with 2.5 mcg/ml fentanyl and group S received 0.0625% bupivacaine with 0.25 mcg/ml sufentanil. Verbal analogue pain scores, need of supplementary/rescue boluses dose of bupivacaine consumed, mode of delivery, maternal satisfaction, and neonatal Apgar scores were recorded. No significant difference was observed between both groups.

**Results:**

Both the groups provided equivalent labor analgesia and maternal satisfaction. The chances of cesarean delivery were also not increased in any group. No difference in the cephalad extent of sensory analgesia, motor block or neonatal Apgar score were observed. Although mean pain scores throughout the labor and delivery were similar in both groups, more patients in fentanyl group required supplementary boluses though not statistically significant.

**Conclusion:**

We conclude that both 0.0625% bupivacaine-fentanyl (2.5 *μ*g/ml) and 0.0625% bupivacaine-sufentanil (0.25 *μ*g/ml) were equally effective by continuous epidural infusion in providing labor analgesia with hemodynamic stability achieving equivalent maternal satisfaction without serious maternal or fetal side effects. We found that sufentanil was 10 times more potent than fentanyl as an analgesic for continuous epidural labor analgesia.

## INTRODUCTION

Labor is a physiologic process but associated with severest form of pain.[1] The goal of labor analgesia is to provide adequate pain relief without causing any maternal and fetal jeopardy. Continuous epidural analgesia is the most versatile and most commonly employed technique, because it can be used for pain relief during labor and for subsequent vaginal delivery as well as analgesia and anesthesia for cesarean section, if necessary.

Various nonpharmacological methods and pharmacological method of providing labor analgesia have been used. Regional analgesia is widely used for providing labor analgesia. Regional techniques present the most flexible, effective, and least depressant options when compared with parenteral and inhalation techniques. Spinal analgesia provides short duration of action. This technique will not be able to provide adequate analgesia for the whole duration of labor. Newer techniques such as combined spinal-epidural, continuous epidural infusions, walking epidurals and patient controlled epidural analgesia (PCEA) are now available.

Fentanyl and sufentanil most commonly used in combination with local anesthetics were found to be effective in providing labor analgesia. Studies comparing fentanyl and sufentanil in labor analgesia are few and give conflicting results. Many investigators have advocated using epidural doses of sufentanil considerably greater than one tenth of the commonly used epidural dose of fentanyl.[2,3]

Hence, we did a study to compare the efficacy between fentanyl and sufentanil combined with low concentration (0.0625%) of bupivacaine for epidural labor analgesia.

## MATERIALS AND METHODS

The study was conducted on 50 full term parturient women of ASA status I and II of mixed parity who were willing for epidural analgesia during labor. The women included in the study had singleton pregnancy with vertex presentation with cervical dilatation of 3–4 cm and had no contraindication to epidural analgesia.

The study was undertaken after Institutional Ethical Committee approval and informed written consent was obtained. Before administration of the epidural block demographic data, parity, gestational age condition of the membrane, cervical dilatation, vital parameters, and fetal heart rate were noted and preloading with 500 ml of ringer lactate was done. Epidural block was given after 3–4 cm cervical dilatation in sitting or lateral position L2-4 interspace with 18 G Touhy’s needle. Loss of resistance to air was used for localization of epidural space. The multi-orifice No. 18 Portex catheter was inspected for its patency and threaded through the needle gently till 3–4 cm length of the catheter is in the epidural space. All the patients received an initial bolus dose of a 10 ml solution containing 0.125% bupivacaine. The patients were randomly divided into two groups according to computer generated randomization.

Group F:-0.0625% bupivacaine with 2.5 mcg/ml fentanyl Group S:-0.0625% bupivacaine with 0.25 mcg/ml sufentanil

The continuous infusion of the study was started at the rate of 12 ml/h after 30 minutes of the initial loading bolus. If analgesia was inadequate, an additional bolus of 5 ml of 0.125% bupivacaine was supplemented after 20 minutes of the initial bolus. The continuous infusion was started using a syringe infusion pump. The infusion was increased or decreased by 2 ml/h and if needed, administered up to two additional 5 ml boluses of 0.125% bupivacaine to provide a pain score of less than 3.

Pain relief was assessed at 20 minutes and then hourly by an observer (who was unaware of the group) using Verbal Analog Scale of 0–10, where 0=no pain and 10=worst pain. Highest level of sensory block, degree of motor block (Bromage Scale), number of additional supplements by anesthetist, total dose of bupivacaine consumed per hour and during the labor, mode of delivery and neonatal Apgar scores were recorded. SpO_2_, heart rate and BP were monitored throughout the labor. FHR was monitored continuously using cardiotochograph. Any adverse effect such as weakness of limbs, hypotension, arterial desaturation, and pruritus were noted and managed if required.

Data were analyzed using INSTAT 3 (GraphPad Software, CA, USA). The data are presented as mean±SD or % or number of patients. Sample size of 50 with 25 patients in each group with power of study 80% to detect a statistically significant difference in the verbal analogue pain score. Two-sided independent Student’s *t*-tests to analyze continuous data and Fisher’s exact test for categorical data. The *P* value<0.05 was considered significant.

## RESULTS

There was no difference between the two groups regarding maternal demography and obstetrical characteristics, duration of labor, incidence of cesarean delivery and Apgar scores [Table 1].

**Table 1:** Maternal demography, obstetrical characteristics, duration of labor, incidence of cesarean delivery and Apgar scores

Parameters (Mean±SD)	Group F (n=25)	Group S (n=25)
Age in years	22.52±2.43	22±2.88
Weight in kgs.	58.52±4.93	57.88±4.41
Height in cms	157.92±5.13	158.12±5.31
Primi/ Multi Gravida	14/11	12/13
Gestational age in weeks	37.96±1.54	37.88±1.31
Cervical dilatation at the start of epidural analgesia (cms)	3.46±0.58	3.76±0.52
Baby weight (kg)	2.64±0.21	2.57±0.19
Vaginal/Forceps/cesarean	22/2/1	21/3/1
Neonatal Apgar score	8.41±10	8.48±10

There was no significant difference in the dose requirement of bupivacaine between the two groups [Table 2]. The number of additional bolus supplementary analgesic top ups were comparatively less in sufentanil group though difference was not statistically significant. No difference in the cephalad extent of sensory analgesia and motor block [Table 3]. The analgesic technique had no significant influence on the mode of delivery [Table 1].

**Table 2:** Dose requirement

Dose (Mean ±SD)	Group F (n=25)	Group S (n=25)
Total dose of bupivacaine (mg)	46.48±6.42	46.64±5.03
Total dose of opioids (μg)	117.42±21.42	12.02±1.98

**Table 3:** Upper level of sensory blockade

Upper level of sensory blockade	Group F	Group S
T6/T8/T10	2/17/6	2/18/5
Modified bromage scale 0/1/2/3	25/0/0/0	25/0/0/0

**Figure 1:**
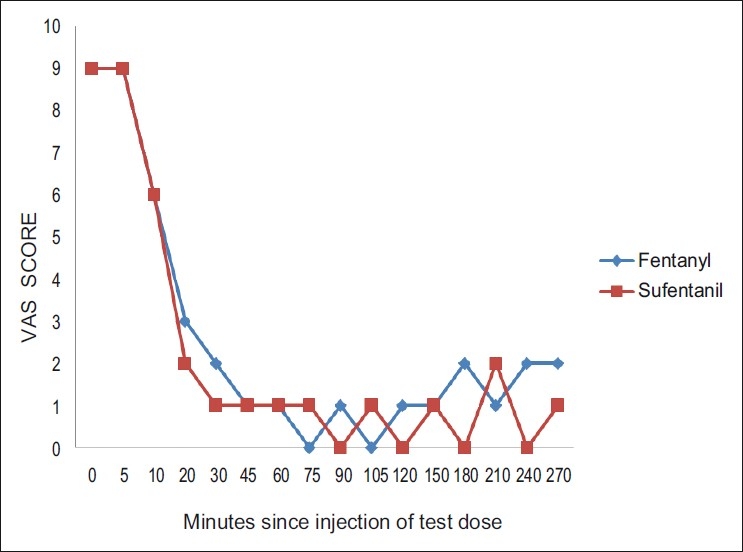
The median visual analogue scale (VAS) score during the active phase of first stage of labor. Analgesia as assessed by the Visual Analogue Scale (VAS) was analyzed on the basis of time relative to the test dose. The figure displays that there was no difference in VAS scores between the groups at any time during the first stage of labor. At the time of inserting the epidural catheter, all the parturients had severe labor pain with median VAS 8–10. After 30 minutes of initial loading bolus, the median VAS was between 0–2. The pain scores during the first stage of labor were comparable without any significant difference between both the groups.

**Figure 2:**
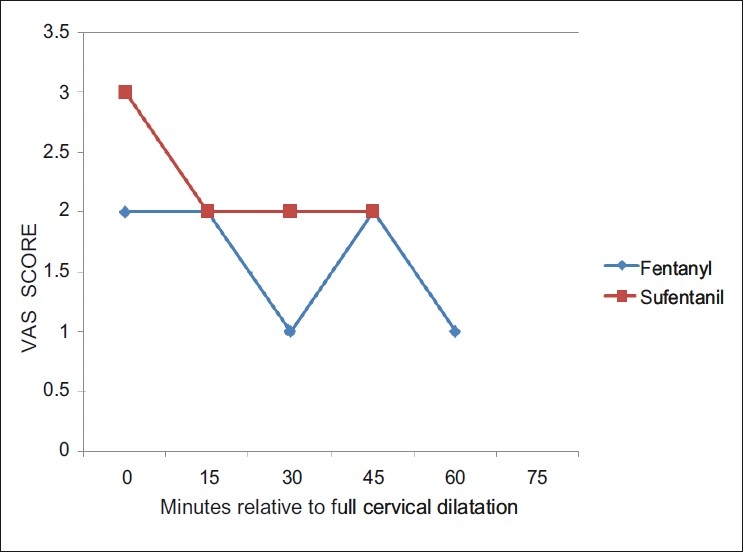
The median visual analogue scale (VAS) score during the second stage of labor. Analgesia as assessed by the VAS during the second stage of labor was analyzed on the basis of time relative to full cervical dilatation. The median VAS score in the second stage was between 1–3 in both the groups. The pain scores during the second stage of labor were comparable without any significant difference between both the groups

The pain scores during the first and second stage of labor were comparable without any significant difference between both the groups. [Figures 1 and 2]. The side effects were comparable in both the groups.

## DISCUSSION

Higher concentration of bupivacaine (0.25%) was used as an intermittent bolus in the past, which resulted in fairly higher incidence of motor block causing pelvic muscle relaxation, fetal malposition, and maternal inability to push and a higher incidence of instrumental delivery. Opioids are commonly being used in combination with local anesthetic drugs through central neuraxial route for labor analgesia. Opioids help in reducing the minimum analgesic dose and concentration of local anesthetic agent given epidurally^4^ and helps in preserving maternal ambulation throughout the process of labor by avoiding motor block. A continuous infusion of a dilute mixture of local anesthetics and opioids offers the advantage of stable level of analgesia, increased maternal hemodynamic stability, less risk of systemic local anesthetic toxicity and slower ascend of level of anesthesia, in case of intravascular or intrathecal migration of the catheter. Both fentanyl and sufentanil[5] were found to have dose sparing effect on bupivacaine when coadministered epidurally.

Both the groups provided equivalent labor analgesia and maternal satisfaction. The chances of cesarean delivery were also not increased in any group. No difference in the cephalad extent of sensory analgesia, motor block or neonatal Apgar score were observed. Although mean pain scores throughout the labor and delivery were similar in both groups, more patients in the fentanyl group required supplementary boluses though not statistically significant.

Our dosing regimen is consistent with various studies,[2,6] which used similar dosing regimen.

A recent report by the American Society of Anaesthesiologist (ASA) task force on Obstetric Anaesthesia concluded that pain relief itself is enough an indication to use epidural analgesia and that cervical dilatation at the time of epidural administration does not impact the outcome of labor.[7] In our study, we appropriately timed to induce epidural analgesia at the early stage after the diagnosis of the active phase of first stage of labor was established.

Other studies[2,8,9] observed no difference in total dose of bupivacaine required between fentanyl and sufentanil group like ours. In contrast, Cohen *et al*.[8] reported that patients receiving sufentanil required less total dose of bupivacaine than those receiving fentanyl.

In the present study, the number of additional supplementary bupivacaine top ups was comparatively less in sufentanil group though not statistically significant different. Cohen *et al*.[8] reported similar finding. However some other studies[11] observed no difference in requirement of number of supplementary bupivacaine top-up injections between sufentanil and fentanyl groups.

The pain scores during the first and second stage of labor were comparable without any significant difference between both the groups [Figures 1 and 2].The side effects were comparable in both the groups, number of breakthrough pain episodes requiring interventions were similar in both the groups. The following studies,[8,11,12] are consistent with our studyand they observed excellent and comparable pain relief with both fentanyl and sufentanil. The side effects were comparable in both the groups.

The duration of labor, incidence of cesarean delivery, and Apgar scores were comparable in both groups likewise as in Connelly *et al*.,[10] Gwen Le *et al*.,[12] Russel *et al*.,[2] Kundialis,[11] Cohen *et al*.[8]

The quality of analgesia was assessed after 24 h of delivery. Patient’s assessment of quality of analgesia and maternal satisfaction was comparable in both the groups

We conclude that both 0.0625% bupivacaine-fentanyl (2.5 μg/ml) and 0.0625% bupivacaine-sufentanil (0.25 μg/ml) were equally effective by continuous epidural infusion in providing labor analgesia with hemodynamic stability achieving equivalent maternal satisfaction without serious maternal or fetal side effects. We found that sufentanil was 10 times more potent than fentanyl as an analgesic for continuous epidural labor analgesia.
